# Modeling Accessibility: Characterizing What We Mean by “Accessible”

**DOI:** 10.1145/3663547.3746344

**Published:** 2025-10-22

**Authors:** Kelly Avery Mack, Jennifer Mankoff, Heather D. Evans, Jesse J Martinez, James Fogarty, Cynthia L Bennett, Aaleyah Lewis, Leah Findlater, Emma J McDonnell

**Affiliations:** University of Washington Seattle, Washington, USA; University of Washington Seattle, Washington, USA; University of Washington Seattle, Washington, USA; University of Washington Seattle, Washington, USA; University of Washington Seattle, Washington, USA; Google New York, New York, USA; University of Washington Seattle, Washington, USA; University of Washington Seattle, Washington, USA; University of Washington Seattle, Washington, USA

## Abstract

Accessibility research has a broad mandate: use technology to make the world more accessible to disabled people. Yet, as a field, accessibility research lacks a clear characterization of what “accessibility” is. Furthermore, it has been historically limited in who is designed for, focusing on specific types of disability and often failing to consider how disability intersects with other identities. We set out to explicate what it means to make something accessible, grounded in the lived experiences of a diverse group of 25 disabled people. From our empirical findings, we develop a process for modeling accessibility. First, an individual assesses their experience of inaccess, specifically, the type of barrier they face, the technology repertoire they possess, and the contextual factors that shape how they address accessibility barriers. Then, having assessed an access barrier, they perform consequence calculus, weighing all available options to achieve access and deciding upon the option that best matches their priorities. We highlight the situated nature of access; people’s identities, contextual factors, repertoires, and priorities all dictate their experience of accessibility.

## 1 Introduction

Accessibility research seeks to make tasks more accessible for a wide range of people and scenarios. Consider three HCI examples that all improve accessibility, but do so in very different ways. When Berke et al. [6] set out to increase access, they focused on how automatic captions could best be formatted to make video content more understandable for Deaf and hard of hearing viewers. Kane et al. [41] addressed early touchscreen inaccessibility for people with vision disabilities by pioneering new screen reader interaction techniques. Finally, Boyd et al. [8] focused on how calming virtual reality environments could increase access for autistic children experiencing sensory overwhelm. Even through three examples it becomes clear: when researchers set out to improve accessibility, there is a wide range of possible approaches and outcomes.

Accessibility researchers do not necessarily have a shared definition of what makes something “accessible”, either theoretically or empirically. Accessibility research has engaged with (and borrowed from) design paradigms from other fields to help motivate increased access (e.g., universal design [45], inclusive design [11]) and developed guiding paradigms for specific kinds of accessible technology design (e.g., ability-based design [74], interdependence [3]). United in aims to increase accessibility, these disparate approaches range in philosophy and execution–perhaps one design should work for everyone [45]; or maybe universal access actually manifests through customization capabilities [74]; or, crucially for this paper, the path to access may vary, but people with disabilities should be central to how access is defined and enacted [3]. As the field develops more approaches to accessible design practice, what accessibility *is*, at its core, remains under-discussed.

Moreover, recent work has shown that accessibility research has been serving only a subset of people with disabilities, necessarily leading to a limited understanding of access. In a survey of accessibility research from 1994–2019, Mack et al. [46] identified an overrepresentation of research into some types of disabilities (e.g., vision disabilities) and an underrepresentation of others (e.g., chronic illness, intellectual and developmental disabilities). Recent work has outlined approaches to better include disability groups such as adults with ADHD [69], people with psychosocial disabilities [61], and chronically ill people [47] in accessibility research. Additionally, recent work has called out a lack of attention to how identities such as race [4, 14, 30, 46], gender [4], and queerness [4, 14] impact disabled people’s experience using accessible technologies. Any understanding of access will not be complete if it is not intersectional, and we center diversity across many axes in our theorizing about access.

In this paper, we argue that a clear conceptual understanding of accessibility is necessary to grow the field. Just as critical disability studies scholarship has progressed in response to models of disability [53, 54, 64], a concrete understanding of *how to model accessibility* can provide designers and researchers insight into how technology operates within the context of disabled people’s lives. Our analysis of participants’ experiences enumerates the range of meanings, goals, and experiences currently combined under the umbrella of ‘accessibility.’ In doing so we aim to expand the domains of access that we study and increase the relevance of our work to people with a diversity of disability experiences.

We are guided by the following research questions:

1. What is the range and variation of ways technology facilitates access?
2. How do an individual’s identities and context impact what it means for technology to make something accessible?

To answer these questions, we conducted hour-long, semi-structured interviews with 25 people who use technology to make their world more accessible. We intentionally sought out participants who, as a group, represented a wide range of disabilities and other identities.

Our findings identify three key dynamics around how technology improves access in participants’ lives. First, we identify that access barriers can take many forms. We name four major types of access barriers we observed in our data: failure point (i.e., a task cannot be completed), usability (i.e., any existing approaches to a task are unsatisfactory), bodymind ^1^ (i.e., existing approaches to a task lead to an undesirable experience for an individual’s bodymind), and future impact (i.e., while a task may be doable in the moment, it will have a negative future impact). Next, we found that our participants used a range of tools in concert to create access. We highlight the importance of considering those tools as a *repertoire* and identify how tools interact: working in *combination* to make a task accessible and serving as multiple *options* to accomplish a single task. Finally, we highlight the ways that contextual factors in an individual’s life–identities they hold, communities they belong to, properties of their technologies, and situational considerations—shape access. These factors inform the available options for an individual’s repertoire and the experience of using technology for access.

From these findings, we create a process for *modeling accessibility*, articulating the nature of access barriers and the process of moving toward access. Our modeling first characterizes a moment of inaccess, highlighting three pieces of information that are central to someone’s experience: relevant contextual factors, the access barrier, and the available tools in their repertoire. Further, it describes a person’s process of deciding how to move toward access by performing *consequence calculus* [47]—outlining possible options to address a specific type of access barrier, weighted based on contextual factors and a person’s repertoire. We conclude with implications for accessibility researchers and designers, including how this process can be used in research and design and other considerations for modeling accessibility.

In summary, this paper contributes:

1. A characterization of accessibility as fluid and dependent on the type of barrier an individual faces, the tools in their repertoire, and contextual factors
2. A process for modeling accessibility, combining our characterization of accessibility with consequence calculus
3. Implications for design that enable researchers and practitioners to understand and design for accessibility as a complex and situated process, better reflecting disabled people’s lived experiences

## 2 Related Work

To enable our analysis of how access functions in the daily lives of disabled people, we turn to three key bodies of work. First, we ground our approach in disability studies theorizing. Next, we identify current theoretical approaches to accessibility within HCI. Finally, we identify gaps in *who* HCI accessibility research considers, turning to intersectionality theory to guide our analysis.

### 2.1 Integrating Perspectives from Disability Studies

We situate our work relative to the disability studies concept of models of disability and with regard to design paradigms from disability studies.

The idea of *models of disability* has been an orienting concept for disability scholarship for decades. Michael Oliver introduced models of disability, naming first the dominant, deficit-based approach to disability as the individual or medical model, followed by a second, activist-minded approach: the social model [53]. In the medical model, disability is seen as a fundamental deficit in an individual—the ultimate goal under the medical model is eliminating disability. The social model, however, treats disability as a natural aspect of human diversity and focuses on removing barriers in the policy, built, and social environments that discriminate against people on the basis of ability. Oliver’s models of disability have been generatively refined, critiqued, and added to since they were first named. These additions include accounting for the multitude of cultural meanings embedded in disability discourses (referred to as the cultural model [20, 68, 73]) and naming how structures of power and interactions between people construct the experience of disability, as described in Kafer’s political/relational model [39]. Yet, simplifying the diversity of disability experience into finite models can draw exclusionary boundaries, and evolutions in theorizing around models of disability were often fueled by advocacy. Feminist disability scholars brought bodies back into disability theory, arguing that locating disability in society, per the social model, erased the embodied experiences that are crucial for conceptualizing how disabled people experience (in)accessibility [23]. Similarly, the political/relational model and activist movements, including disability justice, situate their analysis in sociopolitical systems, highlighting that disabled people’s multifaceted identities and access to resources fundamentally shape what access can mean to them [33, 39]. Accordingly, we aim to articulate a process for *modeling* accessibility, leveraging the analytical capacity of models of disability while emphasizing the situated nature of accessibility.

Disability studies scholars have articulated design paradigms that some HCI researchers have used to guide the design of accessible technologies. HCI has engaged universal [45] and inclusive [11, 56] design practices that encourage designers to create spaces and tools that provide access without requiring special effort on the part of disabled people. A recent critical paradigm, crip technoscience [29], calls upon designers to learn from disabled people’s making practices and center technology design in disability justice activism. While these approaches all provide a structure within which to design accessible technologies, they do not enable a precise articulation of what it means to secure access.

### 2.2 HCI Accessibility Design Paradigms

Accessibility researchers situated in HCI communities have developed a number of accessible design approaches. Each paradigm highlights an important aspect of accessibility, and our work is in conversation with them as we articulate the deeper dynamics that underlie access provisioning.

**Ability-Based Design** emphasizes the need for technology systems to adapt to meet the user, thereby “universally apply[ing] “design-for-one”” strategies [74]. Wobbrock et al. laid out seven principles that position systems to take on this labor of customization with (ideally) minimal disruption to the technology user [74]. While our findings affirm the value of customization, we emphasize that people’s identities, including but not limited to disability, must be considered when designing accessible tools.

Shinohara et al. [65, 67] introduced the need to design for **social accessibility**, emphasizing that technologies must be designed for the social worlds they will be used within. They highlighted that assistive technologies often have a secondary function of marking their users as disabled [66] and articulated processes for designers to attend to social accessibility as they build assistive tools [65].

Bennett et al. [3] translated the disability justice concept of **interdependence** into a framework for the design of accessible technologies. They emphasized that access can be collaborative and centered the autonomy and creativity of disability communities. This framing has been taken up by many HCI researchers, guiding an expanded understanding of accessibility that fosters collaboration and mutualism [16, 31, 49].

Finally, Mack and McDonnell et al. [47] introduce the framework of **consequence-based accessibility** to describe chronically ill people’s access needs. They highlight that people with chronic illnesses often experience a unique type of access barrier, where it is the consequences of their actions, rather than the nature of a task, that makes a task inaccessible.

### 2.3 Expanding Who Is Considered In Accessibility Research

We are dedicated to ensuring that we anchor our modeling in the experiences of a diverse range of disabled people, in line with recent calls to increase diversity among groups studied in accessibility research.

Accessibility research has historically studied a limited subset of the disability community, a trend that has begun to change in recent years. Prior work demonstrates that HCI accessibility research most often focuses on people with vision disabilities, people with mobility disabilities, or people who are d/Deaf or hard of hearing [46]. Recent work has, for example, moved to better include people with psychosocial disabilities [61], chronic health conditions [47], and neurodivergent people [21, 42, 69]. We planned our recruitment to maximize the variety in types of disability represented among participants.

Beyond the disability identities considered, the field has also grown in its consideration of other minoritized identities’ intersections with disability. Disability justice thinking has guided accessibility researchers’ efforts to conduct more intersectional analyses. Principles of disability justice, as articulated by Sins Invalid, a performance collective of mainly queer, trans, and disabled Black and Indigenous People of Color, call for the leadership of the most impacted [33] and have been echoed by many disability justice activists [38, 51, 57, 58]. Accessibility researchers have worked to integrate disability justice analyses into technology design. Harrington et al. discuss the benefits and opportunities that come from considering race alongside disability when designing technologies [30]. Workshops have encouraged the discussion of applying theory from disability justice into accessibility research [71], and many papers have used disability justice principles in their framing (e.g., [3, 49]). Other works focus on needs at the intersection of disability and non-English languages [18, 26] or refugee status [27]. Bennett et al. and Crawford et al. center the experiences of queer people of color in image descriptions and community centers, respectively [4, 14]. We align ourselves with this shift in accessibility research, because access cannot be fully theorized without attending to the diversity of identities that shape disabled people’s lives.

We anchor our consideration of multiple forms of marginalization in foundational Black feminist theory on intersectionality. Building from contemporary Black women’s activism [12] and Patricia Hill Collins’s theory of the matrix of domination [13], Kimberlé Crenshaw proposed intersectionality as a legal framework that articulates the function of multiple forms of marginalization [15]. Crucially, Crenshaw argues that when someone holds multiple oppressed identities, those identities are inextricably linked. We adopt intersectionality as a critical lens for this work, highlighting that disabled people’s multiple identities must be considered from the start to ensure access—they cannot be an afterthought.

## 3 Method

We conducted 60-minute semi-structured interviews on Zoom with 25 participants to understand 1) how people use tools in the process of making their world more accessible and 2) key factors that shape accessibility.

### 3.1 Protocol

Prior to the study session, we coordinated with participants to ensure we could meet their access needs. We provided a range of accommodations—most commonly, we sent interview questions in advance, used Zoom’s automatic captions, and took breaks throughout the session. Two members of the research team attended each interview, with five authors in total conducting interviews. This study was reviewed and deemed exempt by the University of Washington’s institutional review board, and all participants were given a $50 Tango gift card for their time. We asked participants for their consent to record interviews – all but one participant consented to the recording and we instead documented that interview with copious notes.

Interviews focused mainly on 1) how the tools participants use for accessibility operate in their day-to-day lives and 2) how the non-disability identities they hold and communities they belong to shape how they engage with technology. We began interviews by providing participants a sense of what tools were in-scope for our session, which includes tools traditionally understood as assistive but also technologies that provide access despite not being explicitly designed to do so. We then asked participants about their disability identities and the assistive tools they used. Next, we focused on understanding how their tools functioned in their daily lives and how they came to adopt those tools. Finally, we asked participants to reflect on how identities they hold or communities they belong to interact with their experience of accessibility and use of tools. We encouraged participants to consider identities linked to demographics, like race, gender, or preferred language, as well as identities that stem from other relationships or passions, like being a parent or a dancer. Interviews were semi-structured and tailored to each participant. Our protocol can be found in the supplementary materials.

### 3.2 Participants

We recruited participants through US-based and local community organizations that serve people with disabilities, some which specifically focused on one category of disability (e.g., people with vision disabilities) and some which included people with disabilities generally. We also utilized snowball sampling and our personal networks to round out our sample. We recruited people who identified as “disabled,” or as having a related condition or identity that results in accessibility needs in daily life including having a chronic or mental health condition or being neurodivergent. In the screener survey, we also asked participants to optionally share their race, gender, age, and any other facets of their identity they felt impacted their perspective on assistive technology. We selected participants from the pool of those interested by maximizing for variety among disability and other reported identities. In total, we recruited 25 participants. While many participants identified as being neurodivergent or having a chronic or mental health condition, most also held additional disability identities. Participant demographics are summarized in Table 1.

### 3.3 Analysis

Four authors then analyzed AI-generated interview transcripts. All interviews were coded using both the audio recording and AI-generated transcripts, ensuring that AI bias did not confound our analysis. All quotes included in the final paper were checked against original audio recordings to ensure accuracy. To begin the coding process, authors reviewed a subset of transcripts, reading for high level themes. We converged on eight themes including “assistive technology use case,” and “identity impacting tool use.” Then, we sorted each transcript into these eight themes. Each transcript was reviewed by two authors, one conducting an initial sorting pass with the second checking their work. After this stage, authors narrowed the focus of our analysis to six main themes that were most relevant to our paper’s evolving focus.^2^.

We then conducted a deeper inductive or deductive coding pass on the remaining six themes. For themes where we needed to extract a list of information (e.g., types of tools used), we performed deductive analysis. For themes that we needed to analyze more deeply (e.g., identity impacting tool use) one author affinity diagrammed the data, and their work was double checked by another author. Affinity diagrams were presented to the four coding authors for review and discussion. Our results come from a synthesis of our deductive and inductive analyses.

### 3.4 Positionality

The findings in this paper are indelibly shaped by authors’ identities and perspectives. Many authors of this paper are disabled, and our analysis is grounded in that lived experience. Our team of authors include individuals who identify as Black American, Latinx, and White American, and are all based in the United States. Authors have disciplinary backgrounds in computing, design, and disability studies and hold significant expertise in accessibility research. We acknowledge that our understanding of disability, accessibility, race, and other identities is rooted in a U.S. context, based on our collective positionality and experiences.

## 4 Results

Our findings highlight how access technologies function in participants’ lives, and the factors that impact their use. We first provide an overview of technologies participants used and identities that shaped their experiences of accessibility. We then identify four major types of accessibility barriers participants faced: failure point, usability, bodymind, and future impact barriers. Next, we discuss how participants described their intertwined use of, sometimes extensive, collections of assistive technologies throughout their lives, which we term *repertoires*. We conclude by highlighting contextual factors that impacted technology acquisition, selection, and use, emphasizing that participants’ identities greatly impacted if or how well they could use a tool.

### 4.1 Disability and Technology Background

To begin, we highlight the variety that characterized participants’ disability experiences, tool use, and non-disability identities.

Participants in our study held a wide range of disability identities, which led to a diverse range of technology needs. In Table 2, we show a subset of tools used by 8 participants, selected to highlight a diversity of disability experiences. See Appendix A for a more comprehensive list of tools used by study participants. Those with multiple disabilities often needed eclectic sets of tools to meet their needs - for instance the participant in Table 2 R5 used braces to support her wrists while using remote ASL interpreting to access phone calls. Some technologies were useful to people with many different types of disabilities; for example, headphones supported screen reader use in public and let neurodivergent participants control their sensory experience without impacting others. While many participants used tools designed for accessibility, other tools not centered around access such as online grocery ordering, voice-activated speakers, podcasts, and Microsoft Teams played critical accessibility roles as well. While we list only notable tools these participants used for access, some described dozens of tools in their hour-long interview.

Participants shared a wide range of identities they hold and communities they belong to that shaped or were shaped by their technology use (see Table 3), which we discuss in depth in Section 4.4. For example, P9, who is Black, describes needing to code switch while using automatic transcription tools because her mostly Black team is frequently captioned inaccurately. We provide further specific examples of how these factors affect technology use in Appendix A. We highlight that, while demographic identities had a significant impact on participant experiences, so too did identities related to relationships and activities. For instance, P4, who is neurodivergent and focuses better when audio plays in the background, is mindful of the fact that her spouse, who is blind, relies on hearing auditory information clearly.

### 4.2 Characterizing Types of Access Barriers

Participants used technology to address a wide range of access barriers, which we characterize in Table 4. Notably, participants did not only experience access barriers as the inability to do a task without support. Participants described times where access came from improving the experience of performing a task or allowing them to avoid future pain or discomfort. For each type of access barrier we identified in our participants’ experiences, we name it and describe the function of technology in mitigating that type of barrier.

#### 4.2.1 Failure point: a tool makes an impossible task possible.

Participants described that, for some access barriers, there was no practical way that they could complete a task without support. We describe this kind of access barrier as a *failure point*.

For example, P3, who uses a wheelchair, explained *“So [my] wheelchair, obviously, is a necessity. It’s not an option to go away from it.”* Similarly, P17 emphasized *“No matter what, I have to have a screen reader … nothing is gonna be done for me without a screen reader.”* Without these critical tools, P3 and P17 did not have alternative solutions to approach daily tasks like moving about the world or accomplishing tasks at work. P6 found that only some aspects of a task were failure point barriers – she reflected: *“Can I get dressed without [a dressing stick]? Yes. Can I get fully dressed without it? No. So I can put on my shirt and everything but pants. If I didn’t have the dressing stick I wouldn’t be able to get pants on.”* While she could put on a dress and be ready to go outside, any outfit involving pants was a failure point without her dressing stick.

#### 4.2.2 Usability Barriers: Technology changes a quality of the task.

Participants also experienced access barriers when a task could be completed, but not with the qualities they desired. Technology made these tasks accessible by allowing them to perform the task in a way that better aligned with their preferences (e.g., faster, slower, easier). We describe this type of access barrier as a *usability barrier*.

When describing how technology made their world more accessible, participants often emphasized how using technology meant they could complete a task *better*. For P4, fidget toys meant she could *“focus better on listening to somebody,”* P18 often views Google Docs on his phone where access is *“a little better”* than on his computer, and P9 found that captions made them feel like they could *“hear [words] better, even with the volume the same.”* What ‘better’ meant to participants varied across contexts, but it is clear that, without certain qualities, a task is inaccessible.

For some, a more accessible experience meant meeting basic usability characteristics. P16 describes how the quality of performing a task can impact accessibility: *“You could give me a web page that’s perfectly done with your HTML … you labeled everything right. But you happened to use nothing but links and heading level ones, I can’t really navigate that page in any useful way.”* P16 is considering a different type of accessibility than discussed in Section 4.2.1. He can technically consume the content of this hypothetical web page. However, with a poor heading structure and an over-reliance on links, it is time consuming and confusing to navigate, making it unusable and therefore inaccessible.

Another way an access barrier could be addressed was by making a task mentally easier or reducing cognitive load. Several participants used captions to communicate and, for many of them, while communication without captions was possible, it was far more mentally draining. P7 has auditory processing issues related to their neurodivergence, and finds when video call meetings at work do not have captions and multiple people are speaking at the same time, “[it] just feels very overwhelming. Like, to a certain extent, my brain just shuts down… it feels very hard to engage in those spaces, because it all just kind of sounds like garbled noise, and there’s not really a way to translate it.” Captions enable them to more fully participate in meetings because they do not have to devote cognitive processing to decoding audio.

#### 4.2.3 Bodymind Barriers: Technology changes the state of the bodymind.

For many participants, the accessibility of a task depended on the state of their bodymind. An access barrier arose when their bodymind was in an undesirable state (e.g., pained, fatigued, distracted). We name these types of barriers *bodymind barriers*. To make a task accessible, therefore, they needed tools that helped move their bodymind toward a more preferable state.

A key access barrier that participants expressed was needing to complete a task, but feeling deeply uncomfortable or ill while doing so. Technology, then, helped them move from an uncomfortable state to a more comfortable one, managing symptoms such as pain, dizziness, or fatigue. P10, who experiences both chronic fatigue and pain, makes storage systems for his pain relief technology so that it is always close by and easy to access. P9 describes using a variety of tools to manage their pain while performing everyday tasks in their life. Some, like a monitor and chair, encouraged working in positions that would cause less pain, and others mitigated existing pain, like a massage gun or TENS machine.

For others, access barriers took the shape of significant anxiety or emotional distress around a task. P12, who is neurodivergent, has experienced significant judgment around failing to use the “correct” tone in an email and finds that ChatGPT now alleviates their distress while emailing: *“[Without ChatGPT, I would] continue getting in trouble and or written up for my tonality in emails. I would continue to cry more … It becomes a big, like, pain point for me.”* Other participants listed medication as critical to helping them manage their anxiety, depression, or the distress associated with being unfocused (P4, P9).

For many neurodivergent participants, being over- or under-stimulated is an access barrier. P4 works in a quiet office and has found a solution—*“I can’t work in silence … I have my iPad playing [mindless television] all the time [while I’m] sitting at my desk because I have to have that background noise.”* P4 desired increased sensory input, but for others, decreasing sensory input was the goal (P7, P22).

#### 4.2.4 Future Impact Barriers: Technology supports people in avoiding or mitigating future impacts.

Finally, participants described scenarios where access barriers arose based on the future impacts of doing a task. We call these barriers *future impact barriers.* When experiencing a future impact barrier, a person may be able to complete a task (failure point) quickly or accurately (usability) while feeling little to no distress (bodymind), but they may still consider the task inaccessible because they will experience an access barrier later (e.g., pain, inability to complete an important task, etc.). Tools could help participants address a future impact barrier by helping them perform the current task in an accessible way that avoids the future barrier, or by helping them prepare to deal with the future barrier.

Participants who were blind described situations where they chose their approach to making a task accessible to avoid a future impact barrier. For example, most blind participants sometimes used Aira^3^. Although Aira could effectively remove failure point or usability barriers for a variety of tasks, P18 describes it as *“a last resort, because it’s a subscription, and it costs money, and you have a limited amount of time and minutes.”* P19 emphasizes the reality that many blind people cannot afford to purchase more minutes: *“I’m also low income. [Technology companies] know that, but they know because there’s so few competitors they put the pricing at whatever they want.”* Thus, participants treated Aira minutes as a precious commodity; using up their minutes on tasks that could otherwise be made reasonably accessible could leave participants facing future tasks without alternatives. For instance, P16 noted that, while he could use Aira to make sense of his washing machine dial, that would be *“wildly inefficient”*–he instead uses bump dots to mark important settings.^4^ Similarly, P18 saves his Aira minutes for higher-stakes tasks, *“especially when it’s just not screen reader accessible”* or when under time pressure: *“is this the right train … and I only have a few minutes or seconds to figure it out, that kind of last resort thing.”*

P1 cannot always completely avoid or eliminate a future impact barrier, but self tracking tools have given her enough understanding of her bodymind to better predict, avoid, or mitigate the future bodymind barrier. Tracking migraine triggers and useful interventions *has enabled me … to make a lot more choices based on the understanding that, instead of staying [inside] in fear of having a migraine, I can react to them when they happen.”* Self tracking both enabled P1 to avoid future barriers, by developing a stronger understanding of migraine triggers, and prepared her to better address the bodymind barrier her migraines pose when they occur.

### 4.3 How Tools Interact with Each Other: Repertoires

All participants used multiple tools throughout their daily lives, and they often used tools in coordination. Prior work has identified the fact that accessible tools are often not used in isolation [1, 3, 18, 19, 50]. Desai et al. leverage the framing of *linguistic* repertoires to understand the experiences of multilingual captioning users [18]. To understand the set of tools available to participants and how they utilize their tools we introduce the framing of **technology repertoires.** Through analyzing the technologies participants used, we identify two major types of repertoires: many tools working together to provide access to a single task (“combination repertoires”), and many tools that are tailored to different contexts addressing the same task (“option repertoires”).

#### 4.3.1 Combination Repertoires.

For some access barriers, participants’ ideal access solution involved using multiple tools together to address the need, which we term a *combination repertoire.* To engage in in-person conversations, P2, who is deaf and hard of hearing, employs a combination of tools that each provide different types of information that allow for greater communication access when used jointly: automatic captions on her laptop offer both higher accuracy in identifying what words are said and offload cognitive burden; her bluetooth hearing aids offer information on spatial location and speaker identification; and good lighting supports speechreading, which offers more emotional context.

A state-sponsored accessible bus system is P22’s key source of independent mobility in her city, but the bus is loud and sensorially overwhelming. She uses a range of tools to help feel more calm on the bus, including wireless headphones to play music, distracting and calming phone games, and a lanyard full of things she can fidget with. These tools each provide a different form of sensory regulation but work together to make loud spaces more tolerable.

In some cases, participants needed a combination of tools at once because multiple access needs arose from different disabilities that impacted the same task. P1 has symptoms triggered by being outdoors and frequently sprains her ankle. To enjoy a walk outside she can use tools like environmentally protective clothing as well as a cane to meet all of the access needs this task poses. Combination repertoires help us understand how participants secure access in complex situations or when needing to meet multiple access needs at once.

#### 4.3.2 Option Repertoires.

Additionally, participants described having multiple tools that allowed them to address the same access barrier and were useful in different contexts, which we term an *option repertoire.* P4 describes having *“absolutely tons of fidgets that I play with,”* that can help her focus, and when picking which one to use in a specific context: *“the choice [of what tool I use] comes more from what? How am I participating? And who are the people around me that I may or may not be impacting?”* She explains that social context impacts her tool choice, and she selects quieter fidgets when the noise might bother those around her.

Other participants described building options into their repertoire because it made them feel more prepared. For example, there are many apps that aid in non-visual navigation, but they rely on having a charged phone. P18 described how he purposefully plans so that: *“if I can’t do it [with technology], I have plan B, C, and D… if the sun’s out, I could tell you north, west, east, or south by going outside and knowing the time of the day, and not necessarily pulling out my compass on my iPhone.”* Having a technology repertoire that allows participants to complete the same task in myriad ways provided security and confidence that, regardless of circumstance, they would be able to make that task accessible.

Some participants observed benefits from mixing repertoire types. When P19, who is blind, wants to know what is in a photo, she considers the **options** at her disposal: the input of sighted people, paid visual interpretation services, or AI tools. She describes that an AI photo identification app would be most useful when: *“I don’t want to pay for [that photo to be identified], or I don’t have someone available, or it’s a private picture that I don’t want somebody else seeing.”* However, she often then **combines** multiple AI tools to get a more accurate description. This strategy is particularly useful because descriptions are inconsistent–she found that different AI apps *“kind of describ[e] differently between, like different races or ethnicities or skin color.”*

#### 4.3.3 Trade-offs and Gaps in Repertoires.

At surface level, a larger, more complete repertoire might seem ideal. However, participants described challenges in managing a large repertoire and finding tools that would complete their repertoire due to a lack of available options.

Participants encountered significant trade-offs between having few multipurpose tools versus many bespoke tools. For some, an abundance of technologies could be expensive and hard to manage. P16 explained that, when considering acquiring a new tool, he asks: *“how much does it weigh? How much space does it take up? How much of a goofball do you look like carrying a gigantic backpack just to go down the block, because you have all your devices and cords and whatnot?”* On the other hand, P10 celebrated having a wide range of tools on hand that served very specific purposes. P10 is a maker and crafted his house so that he could access his repertoire effectively, including 3D printing custom holders to organize his many tools.

Participants also described ways that their repertoires were incomplete or insufficient. P21 uses a large number of adaptive tools to try to make everyday activities, such as cooking, folding clothes, and drinking from a cup possible for her. Despite the effort she and her occupational therapist have put into finding helpful tools, she still lacks independence in many of these areas. She reflected that *“it’s gonna be really exciting when I find the right tools to help me really succeed in my daily life.”* P21’s repertoire remains incomplete and access barriers persist because no commercial solutions fit her needs.

### 4.4 What Influences Technology Choice and Use: Contextual Factors

A final major driver of accessibility in participants’ lives is *contextual factors*: the characteristics of a person, their tools, or their environment that can influence their experiences of inaccess or moving towards access. Participants described contextual factors connected to their identities (e.g., race, gender, class) and situational context (e.g., location, availability of disability services). Many of the contextual factors we identify are connected to systems of power–forms of marginalization often dictated how our participants could approach access in their day-to-day lives. We identify two major functions of contextual factors: determining what tools participants have in their repertoires and changing their experiences of using those tools.

#### 4.4.1 Contextual Factors Shape Participants’ Technology Repertoires.

First, characteristics of tools, situational context, and identities shaped what tools participants could or chose to include in their repertoires.

##### Technology Characteristics.

Qualities of assistive tools themselves were a preliminary factor in determining their utility. Many properties of technologies that participants saw as important are well-represented in prior literature: performance (e.g., accuracy, efficiency) [22, 40], durability [10], ease of use [2, 6, 10, 52], and system requirements (e.g., battery life, Wi-Fi, portability) [10]. Our participants emphasize the importance of these characteristics, with P19 explaining: *“I live on a sailboat… and so I won’t always have access to the Internet. And so many of these apps like barcode readers with SeeingAI, and these different features rely on the Internet.”* Whether or not a tool could function without Wi-Fi was often the deciding factor in whether or not P19 used it.

##### Identity Characteristics that Limited Tool Options.

Participants who held minoritized identities often had less access to tools or supports, due to pervasive oppressive systems.

Many tools are prohibitively expensive, a reality P20 faces as he figures out how to make his life accessible to him as a quadriplegic wheelchair user. P20 and his partner moved to an accessible apartment with a collection of tools they were only able to purchase with the financial support of family and friends. Still, a bed that could limit how much he needs to be turned in the night remains in storage because their apartment elevator is not big enough to fit the bed and moving to another accessible apartment is too expensive.

Accessible technology availability is not only limited by cost, but also by structures that shape who can access and learn to use those tools. As P8 began to understand themself as autistic, they sought support from local services, only to be turned away because in their area *“you only qualify for services if you’re considered, like moderately to severely [autistic].”* While funded services existed, P8 could not access them because of documentation requirements. Other participants could access well-developed support services, and demonstrate their value. P18 became blind at three years old, and from the time *“they gave me a cane at the age of four”* he received consistent orientation and mobility training. He reflected on the impact of his parents’ dedication to encouraging his independence: *“I attribute a lot of my exploration and experience to that, and having a good support system.”* Participants raised in households with disability stigma, on the other hand, had less access to technology at formative ages. P21 grew up with parents who believed that it was *“very shameful to have a child with disabilities,”*, which has left her to develop her accessible technology repertoire for the first time in adulthood. In these examples, a host of contextual factors including cost, disability services policy, and family beliefs could all keep useful tools out of a participant’s repertoire.

For some participants, available tools did not get added to their repertoire because contextual factors made them functionally unusable. When P2 communicates in English she regularly uses automatic captioning, but the language her family communicates in is poorly supported by automatic speech recognition. Her relationships with her family are impacted by the fact that, without usable captions, *“I don’t have the support I need in this context to maintain touch.”* Safety was also a significant factor that eliminated tools from consideration. P25 worried about being perceived as weak and vulnerable when out in his community, so chose to not use a white cane because *“I’m one that don’t like to be taken advantage of, and I’m not going to invite it to me.”* P19’s spatial context is dominated by the fact that she lives on a boat – unlike many blind people, she cannot use organizational tools that depend upon things staying in a consistent place in her home. Contextual factors, such as language or perceived safety, could make even available and commonsense tools unusable to participants.

#### 4.4.2 Contextual Factors Shape Participants’ Experiences Using a Tool.

Contextual factors also shape accessibility through their impact on the experience of using a tool. While many participants experienced contextual factors that made tool use less comfortable, some found that tools could engage meaningfully with other factors in their lives.

Participants described instances where contextual factors made tools less comfortable to use. P10 is an activist and mindful of his privacy, so, when he can, he only uses transcription tools that do not record or share data. However, when talking to a friend who relies upon transcription tools that store data, he concedes to being recorded because there are no better options. For some, limited technology options do not consider their identities. P5, who is African American, uses braces to manage and prevent injury, but finds that *“the beige or ‘skin tone’ for braces has never fit my skin tone.”* Participants also sought to express their gender identity more fully but were limited by the lack of stylistic variety in apparel that is made to fit people who use wheelchairs (P5) or UV protective apparel (P1). When a tool is not designed with attention to the diversity of disabled people, many are left without tools that match their whole selves. Much of the time, P7 benefits significantly from using noise-canceling headphones for sensory regulation. However, when in a public context, they often forego using headphones because it *“put[s] me at risk of being unsafe and feeling like I constantly have to be vigilant… [to] protect my safety as a queer and trans person of color.”* Especially for people who are multiply-marginalized, technology does not always afford them greater safety when moving through the world.

At the same time, participants also described times where technology use honored or engaged deeply with their identities, communities, and other contextual factors. As she manages a serious skin condition, P24 has found technologies that can connect to her cultural heritage: traditional herbs. She recounted taking *“really strong, bitter herbs for, like, over 10 years”* as a child, and felt that they *“really got me through when Western medicine was not it.”* Being able to engage with a tool connected to her culture is *“just really like soothing for me. Physically and emotionally.”* For P21, her technologies allowed her to build up an identity that had otherwise felt out of reach. After months of meetups where all communication is AAC-mediated she *“feel[s] empowered and enlightened and hopeful for my success as an evolving AAC user and the possibilities for me really becoming a true communicator.”* For P11, tools offered opportunities to further express their gender identity: *“I want my cane to match my outfit when I’m looking hella cute being all trans and loud.”* One of the reasons P12 has found ChatGPT so useful in their daily life is that it uniquely honors their identity. They explain that ChatGPT *“has never misgendered me. Unlike myself, or unlike my friends, like in general … it adjusts everything for me.”* Though it could be more difficult to find tools that aligned with all the contextual factors in participants’ lives, when that alignment occurred, technology use could be a source of empowerment and connection.

## 5 Modeling Accessibility

**Figure 1:**
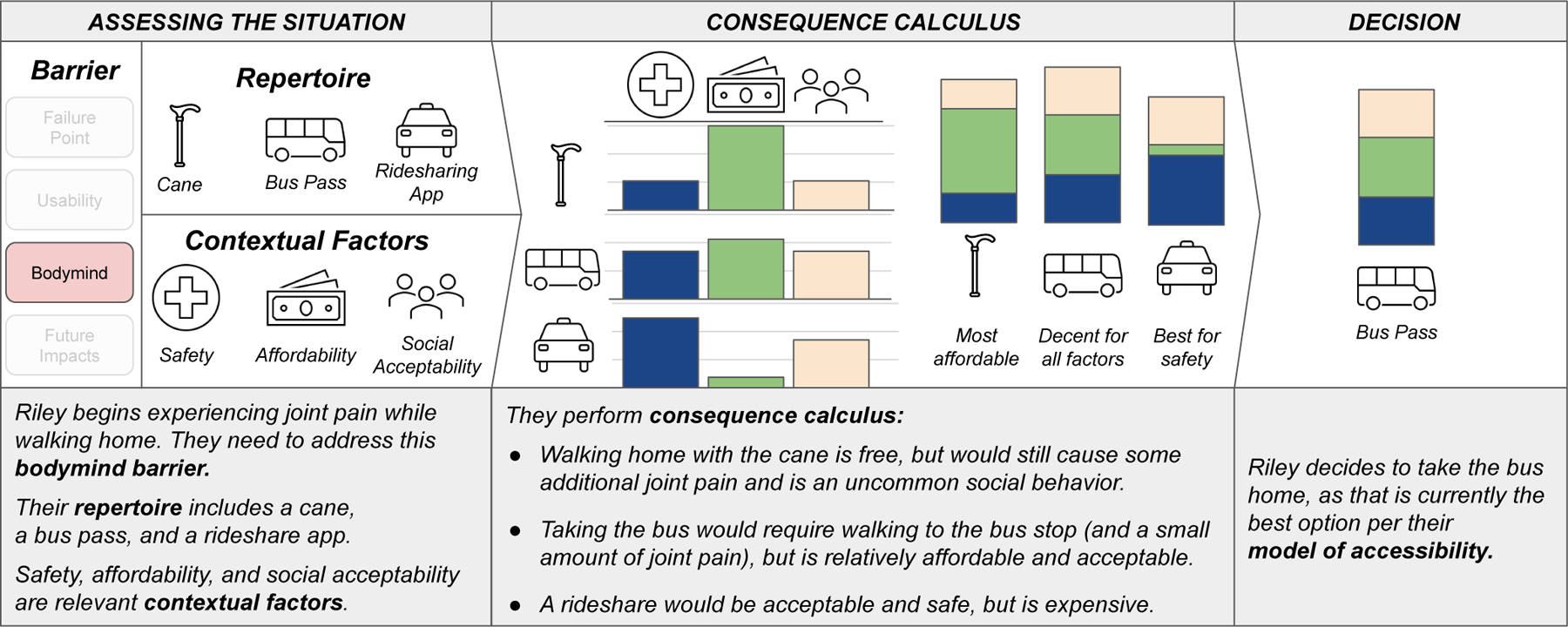
An illustration of the process of modeling accessibility.

Having named the variety of access barriers participants face (Section 4.2), the range of tools they use to address access barriers (Section 4.3), and the contextual factors that shape how they experience accessibility (Section 4.4), we now knit these findings together to synthesize a process for *modeling accessibility.* We articulate the key inflection points for modeling accessibility: describing an access barrier; taking stock of the repertoire at hand; and understanding the contextual factors that shape the repertoire and experience of the access barrier. As demonstrated by the synthetic example of Riley in Figure 1, once a person customizes this generic model (by considering their access barrier, repertoire, and contextual factors), and performs consequence calculus to determine a path forward, they have created a personal, contextual model of accessibility.

### 5.1 Assessment of the Scenario

When someone has an inaccessible experience, there are a multitude of influential pieces of information at play, which we diagram in the “assessing the situation” stage of Figure 1. One of these pieces is a fundamental description of what the access barrier is (e.g., I am feeling too much pain while completing this task, experiencing a bodymind barrier). Another is the tools, or repertoire, available that could address this barrier. And the final, critical information is relevant contextual factors including identities, characteristics of the tools, and other situational factors.

Together, these factors characterize the experience of inaccessibility and are inseparable. For example, the context of a person’s identities can impact how they define their experience of an access barrier. A person who is low income might necessarily scope their repertoire to not include an expensive, motorized wheelchair of any kind, making independent movement a failure point for them. In contrast, someone who can purchase a very slow, old motorized wheelchair may face a usability barrier. Further, what someone fundamentally defines as a barrier might change based on the context or their positionality. P13, who is hard of hearing, often doesn’t view herself as experiencing a communication access barrier at optional social gatherings, since she is a self-described introvert and would prefer not to interact with people.

### 5.2 Consequence Calculus

Having identified the type of access barriers, available tools, and relevant contextual factors, the next inflection point in modeling accessibility is the decision making process, which we call **consequence calculus**. We adopt the term consequence calculus from Mack and McDonnell et al. [47], who define it as a process by which *“individuals determine what is inaccessible to them at a given time based on deeply personal and contextual factors.”* ^5^ Participants describe processes, often second nature, where they consider the tools available to them and the context at hand, identifying the available paths to mitigate their access barrier and selecting the one that best matches their priorities in the moment. Notably, this calculus was limited in instances where users had no or only one feasible option for making a task accessible. Yet, accessibility often required engaging in a complex calculus to choose the optimum of many paths forward. Importantly, the optimal path for an individual is not always the path that looks most obviously accessible—access is often one of many priorities an individual is weighing given the contextual factors surrounding the decision. The emotional experience of using a tool–whether it honors someone’s identities or excludes them–may supersede considerations of performance.

To demonstrate consequence calculus, we turn to P11’s experience deciding which mobility aids to use while shopping. As a person with a chronic illness that limits energy and causes pain, they choose between the tools in their repertoire when going shopping: using a store’s motorized shopping cart, their own rollator, or not using any mobility aid. Each choice presents trade-offs. For motorized carts, they report considering: *“What happens if it runs out of battery? Now I’m stuck in the store.”* Their own rollator is more reliable and is rated to hold their weight, which is not true of all chairs. However, P11 notes that *“when I’m using my rollator, I can’t use a cart because my rollator requires two hands”* and they describe having to expend energy getting it in and out of their car. As someone who has the *“privilege of being an ambulatory user”* they also can choose to do a quick trip without mobility aids— they sometimes decide: *“I know this is going to hurt my body, but I’m going to make it quick.”* In other instances, consequences are differently weighted, illuminating how the motivation for their trip shapes which tool they choose. For example, P11 describes considering: *“am I going there because I need to pick up something for this [activist] event I’m going to? Or am I going there for me?”* Notably, this decision-making process is complex when decomposed, but P11’s embodied expertise makes it something they describe as *“a quick cost-benefits in my head.”*

Following consequence calculus, individuals make a decision about how to move forward in addressing an access barrier and finding a way to complete the task accessibly. We demonstrate the full process of modeling accessibility (assessment through consequence calculus through decision) in Table 5, in which we deconstruct this decision making through four synthetic scenarios derived from experiences our participants described.

### 5.3 Access: A Summative Example

Finally, to demonstrate the richness and fluidity of a person’s experience with access, we model one participant’s experience of accessibility at three different points in time as he developed his repertoire for a single task. This example highlights how one model of accessibility does not necessarily characterize a person’s experience outside a single point in time; a person’s model can change drastically depending on the type of access barrier, their repertoire, and relevant contextual factors.

#### 5.3.1 Experiences with a Limited Repertoire

P3 is a person with an acquired mobility disability that impacts hand dexterity. He enjoys a nice glass of wine, and after his injury he wanted to find a wine opener he could use. At first, his repertoire consisted of only a traditional wine opener (corkscrew), which required *“all the hand abilities which I don’t have.”* He explains that it took *“45 minutes to open a wine bottle… I’ve gone through that a couple of times, obviously it’s not very practical.”*

When modeling P3’s experience at this point in time, we see that his repertoire was limited to a traditional corkscrew. With this corkscrew, he experienced a usability barrier; he could open the bottle of wine, but only after 45 minutes, which he (understandably) described as tedious and therefore inaccessible. Depending on the contextual factors at play on a given night, he might perform consequence calculus and decide to wait for his friend to arrive to open the bottle or, if he’s alone, he might opt for a different drink.

#### 5.3.2 Expanding the Repertoire

P3 desired a faster way to open a bottle of wine himself. Consequently, he tried out other wine opening tools, seeing if there was one he wanted to add to his personal repertoire. Contextual factors shaped the kind of tool P3 is most comfortable trying. When looking for a tool, he explains that he weighs the potential impact of stigma, often feeling that using explicitly assistive tools will make it so that he will *“just be standing out all the time, and I don’t want that”* He reflects that his identity as someone who grew up in a non-American culture with a *“negative connotation and the stigma around disabilities”* likely influences his reluctance to use assistive technologies. This perspective extends into how he chooses tools for his repertoire; instead of buying a tool explicitly branded for people with disabilities, he often seeks out mainstream tools first.

Yet, even after finding a mainstream tool that might be useful, trying new solutions was not always a smooth process. Whether or not he is willing to test out a new tool *“depends on my fatigue level at that moment or day, or how my frustrations have been with doing one or the other in the past.”*

To model P3’s experience trying a new wine opener on a night he is feeling fatigued: he faces a bodymind barrier. He may be too tired to try using the new tool. Since the task at hand is to independently open the bottle of wine with the tool to test how long it takes, relying on someone else to open the wine is not an option. His consequence calculus might point towards postponing the task of testing the new tool to another day.

#### 5.3.3 After Expanding the Repertoire: Access.

After trying multiple options, P3 found a tool *“where you have to just press a button and goes in and just takes out the cork, and that works great for me.”* While using this tool, he finally achieves access he is satisfied with. To model this experience, the barrier is a usability one–opening the wine bottle without this new wine opener is technically possible, but not *practically* possible. However, with this tool in his repertoire, P3’s consequence calculus is simple–he chooses to use the electric wine opener, which does not carry stigma and makes the task easy.

## 6 Discussion

Our findings demonstrate a variety of possible access barriers disabled people may face, highlight the role of technology repertoires in shaping access, and emphasize that contextual factors are central to experiences of accessibility. We have synthesized these findings into a process for modeling accessibility, which articulates the assessment and consequence calculus that allows an individual to move from experiencing inaccessibility to experiencing accessibility. We now connect our findings to prior work and highlight opportunities for design.

### 6.1 Connections to Other Accessibility Paradigms

Our results highlight that accessibility and access provisioning are deeply influenced by contextual factors, regardless of the type of access barrier or technologies used. We compile contextual factors discussed by our participants in Appendix A, identifying identity and non-identity factors. Ours is not an exhaustive list and other repositories enumerate additional contextual factors that impact tool choice [9, 10, 30, 63]. Although the scope of our interviews focused on *technology-supported* access provisioning, Bennett et al.’s interdependence framework also broadens the context in which accessibility provisioning operates [3], highlighting the role other people play in enabling access. Finally, participants’ consideration of social factors further emphasizes the relevance of Shinohara et al.’s paradigm of *social accessibility*[65, 67].

Further, we bring our types of access barriers into conversation with Mack and McDonnell et al.’s “consequence-based accessibility.” They expand understandings of inaccessibility to include situations where someone will incur considerable negative consequences from performing a task, and introduce consequence calculus as a method for managing those consequences. Our characterization of types of access barrier builds on this work, and many of the barriers they describe map across our bodymind and future impact barrier types. We also argue that their formulation of consequence calculus is applicable across types of access barriers and relevant to disabled people beyond those with chronic illnesses.

While we contribute an explicit classification of types of access barriers, accessibility research has been conducted addressing all four types of barriers. We identify prior work mitigating failure point barriers (e.g., [24, 25, 41]), usability barriers (e.g., [6, 32, 44, 72]), bodymind barriers (e.g., [8, 62]), and future impact barriers (e.g., [37, 55]). By explicitly naming these access barriers, we enable retrospective analysis of bodies of work and hope to guide future researchers to a clearer articulation of the access barriers they address.

### 6.2 Design Implications

Our results surface new insights for designers and researchers around 1) identifying new research and design spaces and 2) improving specific tool designs.

#### 6.2.1 PuFing Modeling To Use.

Having articulated a process for modeling accessibility, we envision myriad possible applications. Fundamentally, we envision our modeling process as a method by which accessibility can be more specifically named, understood, and decomposed. While well-suited to fundamental research into accessibility, this process could also support technology designers, policy makers, and people with disabilities themselves. Future work could explore whether our modeling process could be used as a form of structured reflection on disabled people’s experiences of accessibility, either to support their own exploration and self-knowledge or to structure information gathering for researchers, designers, and policy makers. Furthermore, our process highlights how complex accessibility is in disabled people’s daily lives, indicating a need to support disabled people in making sense of and meeting nuanced accessibility needs. Our model could be used to support structured and rapidly changing explications of access.

#### 6.2.2 Identify Under-Served Identity Intersections.

Our results highlight that non-disability identities are tightly intertwined with accessibility. Echoing Hamraie [28], we highlight that if researchers and designers do not consider the range of identities the future users of an accessibility tool may hold, they risk considering only the most privileged and further perpetuating structural inequities. For a tool to be practically useful, it should support a person’s other identities as well as their access needs.

Many existing tools do not adequately consider minoritized non-disability identities, such as being a person of color, queer, or low income. Consequently, some participants felt the need to compromise their identities to use a technology, and some forewent using tools altogether. We highlight opportunities for future research and design to better serve disabled people who hold multiple minoritized identities. Furthermore, participants valued opportunities when their non-disability identities were honored and expressed through their assistive technology–future work should consider how to not only avoid harm but enable joy.

#### 6.2.3 Utilize Barrier Types and Consequence Calculus to Reveal Unsolved Problems.

Barrier types and consequence calculus can provide a new understanding of the problems addressed by existing tools, and can reveal problems that are not adequately covered. Perhaps, when viewing a task through a failure point model, it may seem like a person with a disability can perform the task. But, when viewed through a lens of a usability barrier, it becomes clear that there are no solutions that let them do so quickly or easily. For some barriers, it may be impossible to prevent all negative impacts (e.g., a task may either be fast and painful or very slow but pain-free). In these cases, engaging with individuals’ consequence calculus can reveal which types of support may be most useful.

We now highlight how considering design spaces through the lenses of each of the four types of access barriers we articulate reveals new goals and opportunities.

- Failure point barrier: create a solution that allows a person to accomplish a task they could not otherwise do. Often, a failure point results from a lack of a feasible solution in the problem domain, which can produce a very broad, rich design space. However, designers should be cautious not to create disability dongles [34, 35], as the absence of an existing “solution” could be explained by the problem being insufficiently motivated.
- Usability barrier: create a solution that improves some dimension of performance for a user on a task. This motivates investigation of which dimensions are well-addressed by existing tools, as well as which unaddressed dimensions sufficiently motivate a new tool. Designing for usability barriers highlights that technical but onerous solutions are not sufficiently accessible.
- Bodymind barrier: aid the user in performing a task in a more desirable state (e.g., less pain, improved focus). A first step might be working with disabled individuals to understand the discomfort or difficulty they experience while performing a task. Then, while some solutions might focus on altering a person’s bodymind (e.g., a brace, medication) other solutions may aim to create a more sensorially tolerable environment (e.g., changing the lights, temperature).
- Future impact barrier: make it so that a person can perform this task in a way that avoids or mitigates negative future impacts. Two paths from which to approach this problem space include: 1) changing the original task to avoid incurring the future cost (e.g., avoid triggering a migraine) or 2) mitigating the access barrier that arises in the future (e.g., make future tasks more comfortable to complete with a migraine).

#### 6.2.4 Consider Multiple Sites of Change.

Accessibility research has traditionally designed tools that improve accessibility by changing an individual’s environment or interactions with their environment (e.g., [17, 36, 43]). Yet, we highlight the possibility to design tools that directly or indirectly create access by acting upon an individual’s bodymind. Accessibility and disability studies scholars have traditionally attempted to distance design efforts from an appearance of attempting to cure or fix disabled people [48, 53]. Yet, our participants described the value of changing their bodymind in ways that centered access, not cure. We call on the field to consider ways to design thoughtfully to enable individuals to change their bodyminds as they desire.

#### 6.2.5 Consider New Goals a Tool Can Meet.

Novelty is highly valued when designing and researching new potential accessibility tools. However, attending to the ways that people use tools as part of a repertoire reveals opportunities for researchers and designers to revise assumptions around how novel a tool must be to be useful. Participants’ use of option repertoires demonstrates that disabled people will use different tools for the same access need depending on the contextual factors at play. Therefore, the presence of an existing tool that addresses a specific access need does not necessarily negate the value of more tools to address that need in different circumstances. For example, a solution that accomplishes a task without needing wifi could be a valuable addition to people’s option repertoires, even if they already have tools that accomplish that task when using wifi.

Furthermore, considering combination repertoires can redefine the necessary function of a new technology. If tools are designed to fit within combination repertoires, they may be useful without addressing the entirety of an access barrier. However, researchers and designers must take care to ensure that new tools still meaningfully address access barriers to avoid creating superfluous or incomplete solutions.

#### 6.2.6 Design for Interoperability.

When designing accessibility interventions, researchers and designers should move to consider how tools will operate within an individuals’ repertoire. To do so, tool designers must first understand the landscape of technologies that their target audience likely own and use. Designers should then consider how tools can complement and be used alongside other elements of the repertoire. Furthermore, recognizing the constraints that a tool imposes on a repertoire could increase the possibility of it being practically useful and decrease the odds of abandonment. For example, a tool designed to be used while a white cane user navigates must recognize that a white cane requires one hand to be occupied while navigating.

### 6.3 Limitations and Future Work

Our study has several limitations and identifies opportunities for future work. First, our work is scoped to a western- and US-centric perspective. As global definitions and experiences of disability vary [5, 60, 70], we expect that different facets might come into consideration when modeling accessibility. We encourage future work to consider more globally contextually-specific models of accessibility. Second, we define accessibility in terms of an experience performing a task, whereas there might be situations that are ill-formatted as task-driven (e.g., enjoying the sunset). Third, we acknowledge that the identities of our research team are not fully representative of the participant communities whose experiences we analyze (e.g., disability and racial identities), which may influence how we interpret their experiences with (in)access. Further, our participants’ experiences skewed heavily towards people with neurodivergence, chronic illnesses, or mental health conditions, though many of these participants held other disability identities as well. Our modeling process may disproportionately represent the needs of this subset of the disability community. Fourth, we model accessibility assuming the existence of an already-articulated access barrier. Future work modeling how inaccessibility comes to be would be an exciting complement to this paper. As we have identified the role of contextual factors in shaping the experience of accessibility, we suspect that they are also central to how access barriers arise and are felt by disabled people. Fifth, our interview and analysis were scoped to access provisioning that utilizes technologies. Prior HCI research and accounts of lived experiences of disabled individuals highlight the importance of personal and social supports in achieving access [5, 60, 70]. We emphasize that while these efforts were not in scope for our paper, they are important, and not wholly out of the conversation with our model; social solutions could be well-integrated into consequence calculus and should be explored in future work. Finally, we do not intend for our lists of types of access barrier, repertoires, or contextual factors to be complete or immutable. We list these types, repertoires, and factors that were well represented in our dataset, but we anticipate that by applying our model to more contexts, more will be named.

## 7 Conclusion

Accessibility research has a rich diversity of problems it solves and a range of design approaches, but a limited shared characterization of what “accessibility” means. We interviewed 25 people with a variety of disability identities to understand how tools and other identities they hold impact their experience of achieving access. Through understanding their experiences, we identify three key dynamics that critically influence their experience of accessibility. First, we introduce types of access barriers. We name four demonstrated in our participants’ data: failure point, usability, bodymind, and future impact barriers. Second, we introduce technology repertoires, or collections of tools that people use in combination or as options to improve accessibility. And finally, we demonstrate how contextual factors critically shape access, including a person’s experience, what type of barrier they face, and their repertoire.

From these findings, we present a process for modeling accessibility, providing accessibility researchers and practitioners with shared language to theorize about and design for accessibility. The process starts with a person who is experiencing inaccess assessing the situation at hand: taking stock of the type of barrier, available repertoire, and relevant contextual factors. Then, the person conducts consequence calculus, where they use these types of information to enumerate and weigh their different options. Finally, they decide on the path forward that best suits their needs. Plugging these details into a model can produce a personal, contextual model of a person’s experience with accessibility at a given time. By developing a deeper understanding of how accessibility is provisioned, accessibility researchers and accessible tool designers can identify new accessibility problems to address and create more effective solutions.

## Supplementary Material

Supplementary Materials

## Figures and Tables

**Table 1:** Participant demographic data. All fields were open response text boxes where participants could write their answer. Researchers grouped responses into those shown in the table, drawing from exact participant language as much as possible.

Disability		Race	
Addiction	2	African American or Black	3
Blind or Visually Impaired	5	Afro-Latine	1
Chronic Illness	9	Asian/Asian American	5
d/Deaf or Hard-of-Hearing	5	Mexican, Latin American, or Latinx	3
“Disabled” Generally	5	Multiracial	4
Intellectual or Developmental Disability	3	Person of Color	1
Mental Health Condition	7	South or Southeast Asian	2
Motor Disability	9	White	12
Multiple Disabilities	14		
Neurodivergent	11		
Age		Gender	
18–25	4	Female/Woman	11
26–35	9	Genderqueer	2
36–45	4	Gender Non-conforming	1
46–55	4	Male/Man	6
56–65	1	Nonbinary	5
66–75	2	Trans Woman	1

**Table 2:** Notable tools used by 8 participants, selected to highlight diversity in disability identity and tool use. To preserve anonymity and decouple this more specific and potentially identifying data, we refer to these using row numbers (i.e., rather than participant identifiers).

Row #	Disability Type	Notable AT
R1	Neurodivergence	Podcasts, Fidget Toys, Headphones, Computer Games, iPad
R2	Motor, Chronic Illness, Neurodivergence	Wheelchair, AAC, Eye Gaze Detection, Adaptive Gaming Console
R3	Motor, Chronic Illness	Grocery Ordering Service, Portable Ramps, Grit Freedom Chair Outdoor Active Wheelchair, Dressing Stick, Smart Home Devices
R4	Motor, Chronic Illness, Mental Health, Neurodivergence	Antidepressants, Adderall, Captions, Elevated Second Monitor, Earplugs
R5	d/Deaf/Hard of Hearing, Motor, Chronic Illness, Mental Health, Neurodivergence	Brace, Lyft, Translation Apps, Video Remote Interpreting, Microsoft Teams
R6	Vision Disabilities	Audible Pedestrian Signal, Aira, Braille Display, Crosswalk Tactile Bump Mats, Guide Dog, Seeing AI, Headphones
R7	Vision Disabilities, Motor, Intellectual/Developmental, Neurodivergence	AAC, Dragon Dictation Software, Yoga Mat, Screen Reader, Adaptive Cooking Utensils
R8	d/Deaf/Hard of Hearing, Chronic Illness, Mental Health, Neurodivergence	Non-Western Medicines, Ancestral Herbs, Google Translate, Wiktionary, Transcription

**Table 3:** Participants identities and communities they belong to that shape how they use AT.

Identities Related to	Identity Factors
Demographics	age, class, disability, education, ethnicity, gender, income, language background, place of residence, sexuality, race, religion, size
Communities or Relationships	disability community member, family member, friend, partner
Activities	being an advocate, a professional, a hobbyist, an athlete

**Table 4:** Four types of access barriers, the goal of a tool in mitigating each barrier, and examples of participant experiences navigating these barriers with their tool.

Barrier and Goal of Tool	Example
**Failure Point** Tool makes a task that is not possible for a user possible by providing support.	*“So I can put on my shirt and everything but pants. If I didn’t have the dressing stick I wouldn’t be able to get pants on.”* - P6
**Usability Barrier** Tool helps the user perform a task in a way that is more aligned with their preferences (e.g., faster, slower, easier, harder).	*“If sighted people [are] ordering something, it probably takes like 5 minutes –could end up taking me like 30 minutes, or 20 minutes … longer, always longer. Making [ordering] quicker, probably would be something I would ask for.”* - P17
**Bodymind Barrier** Tool helps adjust the user’s bodymind to a more preferred state (e.g., more focused, less pain).	*“If I’m getting dizzy [while watching a video], specifically like, if I’m getting nauseous … I’ll still turn on the captioning. But I might just choose to put on headphones instead, and then kind of avoid looking at the screen instead.”* - P8
**Future Impact Barrier** Tool helps provide information that makes dealing with or planning for future barriers more possible.	*“The one I use the most is this 10 minute timer, an hourglass timer, because I don’t take my phone in the bathroom, and I shower, and also, I can’t hear … I really lose, like all sense of time … sometimes showering makes me not feel good and like if I’ve been showering for way too long, like I need to sit down afterwards, and so that [hourglass timer] gives me like a check on [time].”* - P2

**Table 5:** Four synthetic scenarios deconstructing AT decision-making by: (1) illustrating access barriers; (2) identifying barrier types, contextual factors, and AT repertoires; (3) applying consequence calculus; and (4) arriving at an access choice to complete tasks accessibly.

Benji, who is non-speaking	
**Access Barrier**	When going on a first date, Benji needs a way to communicate, without relying on support from her usual communication assistant: her mom

**Identify**	**Type:** Failure Point **Contextual Factors:** Social expectations; Prioritize independence **Repertoire:** AAC device

**Consequence Calculus**	1. Benji could suggest going to a movie, where they would not communicate much; 2. Benji’s mom could come along to facilitate communication; 3. Benji could use AAC to communicate with her date

**Eventual Access**	Option 3 is by far the best option for Benji–she wants to get to know her date and does not want her mom along
**Jordan, whose left leg is amputated above the knee**	
**Access Barrier**	Jordan’s current prosthetic makes walking very slow, and she is looking to upgrade to one with a powered knee, allowing her to walk faster

**Identify**	**Type:** Usability **Contextual Factors:** Jordan is a Black woman; She is a lawyer and has a dress code at work; The upgraded prosthetic she is looking at only has a pale beige cosmesis*; She could forego a cosmesis, but it will look bionic **Repertoire:** Current prosthetic, New prosthetic with powered knee, pale beige cosmesis

**Consequence Calculus**	1. Jordan can continue using her current leg, which matches her skin tone, but is tedious to walk in; 2. Jordan could get the upgraded leg in beige, which will not honor her racial identity and will make her feel self-conscious while she wears it; 3. Jordan could forego a cosmesis, and have a more obvious prosthetic leg

**Eventual Access**	Jordan chooses option 3– having a leg that allows her to keep up with her friends while working is worth it, and, while she does not enjoy how obvious it is that she is an amputee, it honors her identity as a Black woman better than pale beige would
**Alex, who is Deaf**	
**Access Barrier**	Alex is on a road trip with their friends, and they just walked into a loud restaurant – after a long day in the car, communication is cognitively overwhelming

**Identify**	**Type:** Bodymind **Contextual Factors:** Alex and their friends are notably queer; The restaurant is in a conservative, unfamiliar area; They and their friends know ASL; It’s been a long day, and everyone is tired and ready for food and bed **Repertoire:** Hearing aids; Automatic captions; DoorDash

**Consequence Calculus**	1. Everyone in the group could sign through dinner, but they already feel very out of place as queer people in this restaurant; 2. Alex could suffer through dinner, overwhelmed and not able to join in conversation; 3. They could go to their hotel instead and order dinner on DoorDash

**Eventual Access**	Alex chooses option 3 - asking their friends if they wouldn’t mind driving back to the hotel and ordering DoorDash instead – everyone agrees and opts for a quiet night in
**Juan, who has a visual processing-related chronic health condition**	
**Access Barrier**	Juan’s team at work is sitting down to read a printed out document – he will be able to read it visually, but within the hour, he will be very dizzy and nauseous

**Identify**	**Type:** Future Impact **Contextual Factors:** Social acceptability: Juan will be notably different than his coworkers if he doesn’t read visually, and instead puts in headphones to listen to a text-to-speech (TTS) tool; Time pressure: everyone will be reading this document in 10 minutes or less; Juan has high familiarity with people on the team from working on a past project together. **Repertoire:** Headphones; Laptop with TTS; A PDF of the document; A printed copy of the document

**Consequence Calculus**	1. Juan can visually read the document in full and probably won’t be too dizzy or nauseous before the meeting is over, but the rest of the day will be hard; 2. Juan could skim the document, maintaining social acceptability and not getting too dizzy, but he misses out on potentially necessary content; 3. Juan could pull out his laptop and read the document using TTS and headphones.

**Eventual Access**	In this meeting, which includes only teammates he’s worked with for years and no clients, Juan chooses option 3–his team understands his access needs by now, minimizing his concerns about social acceptability

* A cosmesis is the final covering on a prosthesis which is meant to look more socially acceptable and allow for better gripping ability [7].

## Footnotes

1 Bodymind is a concept introduced by Margaret Price and quickly adopted by many disability scholars and activists [59]. As defined by Sins Invalid, it refers to: “the relationship between the human body and mind as a single integrated entity. This term is used instead of saying ‘body and mind’ to affirm the reality that our minds and bodies cannot be separated” [33]

2 At the end of analysis, authors reviewed the two unanalyzed themes to ensure we did not miss relevant data.

3 Aira is a visual description service that allows users to connect to human visual describers using their phone’s camera.

4 A type of durable adhesive dot useful for creating tactile indicators

5 Paymal and Haywood [55] have also explored how consequence calculus illuminates how people choose technologies, specifically in the case of people with ME/CFS

## Appendix

In the following tables, we provide supplementary examples of 1) identities impacting tool use, 2) non-identity contextual factors impacting tool use, and 3) examples of the diverse range of tools and technologies used by people with different disabilities.

**Table 6:** Instances demonstrating how identity factors related to demographics, community, and activities impact AT use.

Identity Category	Identity Factor	Example Participant Quote
Demographics	Age	*“When you think of hearing aids and pacemakers … the typical demographic that uses them is much, much older … When I was 12 years old getting hearing aids sucked. I hated the idea of it … There was that sense, ‘this is gonna be really othering or really alienating.”‘* - P2
	Class	*“My class background affect[ed] my use of technology … I grew up with parents who were both very highly educated, in a traditional sense, and also worked as engineers in Silicon Valley. So we always had computers growing up. So I would say in an indirect way, that’s also led to me being very comfortable using [the] Internet and computer to look for resources.”* - P8
	Ethnicity	*“My parents’ own history of poverty, and their mentality around money has also, and I think, to some extent, it might be kind of a common experience among Chinese immigrants or Asian immigrants, even if they have money, behave as though they’re still living in scarcity. So, there’s this component where maybe I could afford, for instance, to buy some software. But I wouldn’t do it, because it’s something that I was not raised to think that was necessary.”* - P8
	Gender	*“I do change my gender presentation based on how anemic I am because I have historically received more street harassment when I dress more butch and so I tend to dress more femme when I am more anemic, because I find that I am less likely to find myself in surprise situations where somebody wants to be physically aggressive towards me.”* - P1
	Income	*“I do think there are also great ones like Be My Eyes, [which] is free. … Then you have paid services like Aira, where it’s a trained agent on a video, which is great, but they’re quite expensive. And it’s like, I’m not paying for their program because I can’t afford to pay for it for the times that I might use it … I don’t have that disposable income for access.”* - P19
	Language	*“Automated captioning is not available for a lot of cultural heritage languages … Zoom and Ava [don’t] work for the other languages that I speak.”* - P24
	Residence	*“We [U.S. residents] have so many options for accessibility tools. But there’s so many countries that don’t. I went to Jamaica and … my Deaf friends were shocked to react when I stated that the Deaf children in Jamaica didn’t have [videophones] and they only have one certified interpreter in the rest of Jamaica island.”* - P15
	Queerness	*“I also with, my gender identity and stuff, sometimes [an AT’s style] just doesn’t match, like I want. I want my cane to match my outfit when I’m looking hella cute being all trans and loud, like cool, I got a purple cane to go with a wedding outfit of a suit and tux I had at 1 point, because a purple cane would match it perfectly.”* - P11
	Race	*“As a Black person, I am a little bit wary about some AI tools. … I use some at work. But I still limit what I interact with.”* - P9
	Religion	*“I grew up with a Catholic mother … there [were] a lot of beliefs around how prayer and priests could cure me out of this very quote unquote unfortunate disease.”* - P24
	Size	*“If I’m going to a party or something, and maybe it’s outdoors and they have those plastic lawn chairs, there’s more odds that I can break that, because it’s not going to be weighted for me. Or if I’m going to the doctor’s office or something that has seating but all the chairs have arms, now I’m having to squeeze my butt and my hips into this very defined space.”* - P11
Communities & Relationships	Disability Community Member	*“I do use my AAC device at a group called [AAC Group Name]. … We meet up and we use our devices for communication. So that’s a special community for that.”* - P21
	Friend	*“As far as conversations with friends—personal use. I prefer Google Chat. It’s a free service rather than Zoom. Zoom limits you to 40 min unless you pay. So I prefer Google Chat.”* - P15
	Partner	*‘I have a sighted spouse … I used to do all the laundry in the past for a lot of years when I had a manual machine, a more analog type machine. But now it’s a little more her responsibility now that we have this machine.”* - P16
Activities	Hobbyist	*“I would be an artist, you know. … I felt like I had those wants and talents, but couldn’t really express them until, you know, various technologies made certain strides.”* - P18
	Athlete	*“I quite honestly looked for a grant for the GRIT chair before we had even saved up money to remodel the bathroom so I’d have somewhere to shower. So it does sort of drive my thinking. But for me I’ve always, for the most part, been an outdoor athlete.”* - P6

**Table 7:** Instances demonstrating how non-identity factors related to social contexts, spatial contexts, and institutional supports and barriers impact AT use.

Non-Identity Category	Non-Identity Factor	Example Participant Quote
Social Contexts	Who I am Interacting With	*“[It depends if I’m communicating with] someone who I am like, really familiar with speaking with and like, I’m usually like able to communicate with them about captions. And we have like established processes. Or maybe they know sign right?”* - P2
	Social Perceptions	*“I’m a bit resistant to using assistive technology. I think it also comes from the negative connotation and the stigma around disabilities that I grew up with. So, the culture and the society kind of views disability in a … very negative way.”* - P3
Spatial Contexts	Location	*“At home, I definitely have more access, like, my massage tools are definitely kept at home and stuff. But I do have a small massage roller that I carry in my purse when I’m at work and I have [an] arnica oil that I use that I can bring with me to work and stuff. I can do some limited stretching at work as well, but definitely have more access at home to really care for myself.”* - P9
	Environment Conditions	*“There are people who can’t work with noise. I can’t work in silence… I have to have that noise… when I was in when I was in grad school, and I would be sitting in a coffee shop because I had the coffee shop going around me.”* - P4
	What’s Available in the Environment	*“We didn’t have access to phones at the time [while traveling]. So basically [I] was writing notes back and forth with English to family members who are writing in Spanish.”* - P15
	What Can Change in the Environment	*“[As someone who is COVID conscious, when] anyone comes into my apartment, I am always masked, and [I] ask them to mask. I have several HEPA air purifiers in my apartment. I will open the windows for ventilation.”* - P7
Institutional Supports or Barriers	Bureaucracy and policy (Support)	*“With my case worker, I put in [a request] for … these special blinds, and you need the Alexa to go with it, so I put in a request to get those special blinds.”* - P23
	Bureaucracy and policy (Barrier)	*“[I reached out to see what support] my local regional center would provide. But I was told … you only qualify for services if you’re considered like moderately to severely [autistic] I think they use some kind of like functioning label or something like that. And so I was just like, okay, I don’t think I’m going to qualify for this, because, like as it is like people already, don’t believe that I’m autistic.”* - P8

**Table 8:** A selection of AT used by participants across disability type.

Disability Type	AT Used
Blind or Visually Impaired	AIRA; Audiobooks; Audio Labeling Pen and Stickers; Audible Traffic Signals; Be My Eyes; Beep Ball; Braille Display; Braille Reader; Braille Writer; Bump Dots; Bus App; Curb Cut; Doordash; Eye Medicine; Guide Dog; Instacart; Jaws; Lyft; Magnifier; Meta Glasses; Seeing AI; Smart Home Devices; Soundscape; Tactile Bump Mats At Crosswalks; Talking Alarm Clock; Talking Kiosks; Uber; Uber Eats; Voiceover; White Cane; Zoom
Chronic Illness	Bar Stool; Binaural Tones App; Car Window Tint; Clothing; Custom Orthotics; Disabled Parking Placard; Elevated Second Monitor; Elevator; Ergonomic Laptop Stand; Escalator; Google Suit; Homeopathy; Iron Infusion; Mask; Medication; Motorized Shopping Cart; Non-Western Medicines; Online Medication Refills; Pacemaker; Shopping Cart; Sunbrella; Tinted Sunglasses; Word; Hot Water Bottle
d/Deaf and Hard of Hearing	Bluetooth Hearing aid; Captions; Google Docs; Google Meet Background Google Translate: Noise Filter; Transcriptions
Intellectual or Developmental Disability	AAC; Bathtub Transfer Pole; Big Marker; Dragon Dictation Software; Pillow; Railing; Yoga Mat
Mental Health Condition	AAC; Antidepressants; Google Calendar; Google Docs; Microsoft Word Templates
Motor Disability	Access Bus; Adaptive Gaming Controller; Cell Phone Mount; Collapsible Motorized Wheelchair; Dictation Software; Grocery Ordering Service; Grit Freedom Chair; Keychain Rings; Pillow; Portable Ramp; Predictive Text; Smart Home Devices; Triangular Paint Brush; Uber; Voice Controlled Bidet; Water Bottle with Long Straw; Wheelchair Pants; Wheelchair W Joystick; Wide Doorway; Wine Opener with Push Button
Neurodivergent	AAC; Adderall; Alexa; Calendar and Scheduling Apps; Calm Strips; ChatGPT; Clothes; Earbuds; Fidgets and Stim Toys; Gaming Apps; Grammarly; Google Calendar; Ipad; Kanban Board Software; Lanyard; Lights; Music; Noise Cancelling Headphones; Notes App; Pillows; Podcasts; Smart Home Devices; TV Shows; Zoom
