# Supplementary material for: Modeling Accessibility: Characterizing What We Mean by “Accessible”
Source: ASSETS. Author manuscript; Available in PMC 2026 Mar 10. (PMC12969287; doi:10.1145/3663547.3746344)
Supplement: Supplementary Materials [file NIHMS2121179-supplement-Supplementary_Materials.zip › Interview Protocol Clean.docx]

# Semi-Structured Interview Protocol

As a reminder, we want to hear any “more traditional” technologies you use like canes or screen readers, but we’re also really interested to learn how technologies that are less branded for access help you too, like if Google Home, Candy Crush, or a really well insulated water bottle is really integral to your access in life. We understand that social supports are often a critical part of access provisioning and we would like to hear about those interactions if they involve technology.

# Part 1: Access Needs and Disability Background

To get us started, could you tell us about your experiences with disability, Deafness, chronic health issues, mental health, neurodivergence, or anything that you feel falls into that general umbrella?

Thank you for sharing – we’re going to jump right into thinking about the kinds of tools and technologies you use to make the world more accessible for you. As a reminder, we’re interested in technologies that might traditionally be associated with accessibility, like wheelchairs, as well as any medical tools you use or everyday objects you find have surprising accessibility uses. Could you talk us through your assistive tools and technologies?

We’re now going to talk through how you use these different tools and technologies.

# Part 2: AT Use

*Repeat 2-3 times:*

*Aim to ask about three access needs/technologies, perhaps ideally 1. Traditional AT 2. Medicalized AT, and 3. Non-traditional AT*

- Let’s start with <AT>:
- Think about the most recent or a notable time when you were using <AT>. Tell us about what this tool made possible for you.
  - It sounds to me like <AT> helps you < meet access need>. Can you think of any other tools you use to <meet access need>?
    - Repeat as needed if <AT> meets multiple access needs
  - *Follow up asking about contexts (e.g., I’m hearing that you use X at home, does that change when you’re out and about?))*

*Ask at least one time throughout the above loop of interview:*

- How did you come to select <TOOL>?
  - *Follow up:* if not mentioned – are there other tools you considered using to meet this AN or ones you tried that didn’t work?
- Tell me about a time when it was HARD/more complicated to use x
  - Why was it hard?
- Can you tell me about when you started using <AT>? What was that experience like - how did it feel?

*Optional Follow Ups:*

Can you tell me about a recent or memorable experience about how you acquired one of your assistive technologies? Can you tell me about how you ended up finding it and learning how to use it?

- How did you find out about the different ATs you use/have used in the past?
- *Be ready to probe about things like insurance, longevity, price, social acceptability*
- *Also ready to probe about what alternates they considered but did not adopt*

Could you tell us about an assistive technology that you use differently than you used to?

- Follow up if they don’t have a specific answer – Or, could you tell us about a tool you use now that you didn’t use in the past? What made you take up that tool?
- Probe for why, what changed, if changing access needs have played a role
- Probe for are there broader trends in how AT use has changed over time?

Are there any other assistive tools or technologies that you use frequently that we haven’t discussed so far? (If there are many, prioritize the ones that are most important to your life).

- If it didn’t come up already: are there any AI tools that you use for access purposes?

Having talked about a lot of different technologies you’d use, I’d love for you to reflect with us – how does technology make your world more accessible? What does it do for you?

# Part 3: Identity and Contextual Factors

Now that we’ve discussed a lot of the assistive technologies you use and the contexts they are useful for you in, we’re interested in thinking with you about how facets of yourself and the communities you belong to, other than your <disability> shape the kinds of tools you might choose to use or how it feels to use different assistive technologies. Before we jump into specific questions, I’ve heard you mention that you are <a nonbinary person, white, a dancer, etc>. Are there any other aspects of yourself or the communities you’re part of that you want to share with me, especially any that shape the kinds of accessibility technologies you use?

<Curate a list of mentioned identities>

Thank you so much for sharing those – I’m now going to ask you a couple questions about a few of those identities, first up:

<LOOP through at least 2 identities/communities, perhaps one with a clear demographic connection and one that is more activity oriented? Pay attention to both research goals and any emphasis participants stress when doing so>

Could you tell me about a time when your assistive technology use felt connected to, in conflict with, or driven by your <CURRENT IDENTITY/COMMUNITY>?

- Examples if needed: for instance, we’ve talked to folks who found that the clothes they needed to wear to protect themselves from the sun really don’t match their gender identity, people of color who found that the beige often used in assistive tech design never matched their skin tone, people who used captions but found that automatic captions didn’t work for the language their family speaks.
- *Follow up:* Can you tell me about a time when your experience achieving access using your accessibility technology came at the cost of [honoring/preserving] the rest of yourself or the way you like to show up in <community>?

Now we’re going to brainstorm a bit more, we can throw away practicality and some fun imagining. Earlier we were discussing how you use <AT>. Could you think of ways that <AT> could be differently designed to really mesh with your sense of yourself as <identity>?

<loop end>

- We’ve talked about specific aspects of yourself, and before we wrap up, I want to take a moment to reflect on your whole self.
- Overall, how, if at all, do you feel like parts of yourself other than your disability, shape the kinds of assistive technologies you feel comfortable using?

# Closing

We’re pretty much at the end of the interview – I just want to leave space for any burning thoughts that you want to share before we wrap up?

Thank you so much for your time today, we really enjoyed learning from you. As we mentioned in the recruitment process, we will be compensating you $50 for your time. This will come in the form of a Tango gift card that will be sent from <email>. It tends to take a week or two, please follow up with me if you have not received it after 2 weeks. We want to let you get back to your day, but are happy to stick around if you have any questions.
